# Phenotypic and Differential Gene Expression Analyses of Phase Transition in *Oedaleus Asiaticus* under High-Density Population Stress

**DOI:** 10.3390/insects13111034

**Published:** 2022-11-08

**Authors:** Na Guo, Hongyue Ma, Haibin Han, Feng Yan, Haiyan Gao, Yuanyuan Zhang, Shujing Gao

**Affiliations:** 1 Institute of Grassland Research, Chinese Academy of Agricultural Science, Hohhot 010010, China; 2 Research Center for Grassland Entomology, Inner Mongolia University for Nationalities, Tongliao 028000, China; 3 Forest Pest Management and Quarantine Station of Ordos, Ordos 017010, China

**Keywords:** *Oedaleus asiaticus*, high-density population, phenotypic plasticity, transcriptomics

## Abstract

**Simple Summary:**

*Oedaleus asiaticus* (Bey-Bienko) is one of the most dominant locust species in grassland and pastoral areas in Inner Mongolia of northern China. It is highly abundant and usually makes up more than half (sometimes even up to 90%) of the local locust community in locust outbreaks. Locust aggregation is a prerequisite for a locust outbreak, requiring phase change from solitary to gregarious individuals. In this study, we used Illumina sequencing technology to screen 3911, 7478, 3142, and 1852 differentially expression genes (DEGs) in *Oedaleus asiaticus* during phase transition after 1, 3, 5, and 7 days of high-density treatment, respectively, and recorded the transition from green to brown individuals in different stages. The change in expression patterns of *JHAMT*, *JHEH*, *DIB*, *HPD*, *TAT*, *PAH*, *DDC*, *CSP*, and *TO*, which are the key genes of phase transition relevant metabolic pathways, at different stages of the phenotypic transformation suggests their regulatory role in the phenotypic process. This study improves the scientific understanding of phase variation in locusts; the learning can be applied to other insects.

**Abstract:**

The high-density-dependent phase change from solitary to gregarious individuals in locusts is a typical example of phenotypic plasticity. However, the underlying molecular mechanism is not clear. In this study, first, *Oedaleus asiaticus* were treated with high-density population stress and then analyzed by Illumina sequencing on days 1, 3, 5, and 7 of the body color change to identify the stage-specific differentially expressed genes (DEGs). The KEGG pathway enrichment analysis of the identified DEGs revealed their role in metabolic pathways. Furthermore, the expression patterns of the nine key DEGs were studied in detail; this showed that the material change in locusts began on the third day of the high-density treatment, with the number of DEGs being the largest, indicating the importance of this period in the phase transition. In addition, the phenotypic change involved several key genes of important regulatory pathways, possibly working in a complex network. Phenotypic plasticity in locusts is multifactorial, involving multilevel material network interactions. This study improves the mechanistic understanding of phenotypic variation in insects at the genetic level.

## 1. Introduction

Phenotypic plasticity, the ability of a single genotype to produce different phenotypes, is an important aspect of insect adaptation to different environments [1,2]. It has been reported in a variety of insects; however, locusts have received the most attention for their phase transition between the solitary and gregarious phases, causing severe crop loss [3]. Phenotypic plasticity in locusts is known as phase polyphenism, in which local population density affects the expression of various behavioral, physiological, and morphological traits [4]. In recent years, several regulatory mechanisms related to phenotypic plasticity in pests have been identified, including hormone regulation, differential gene expression, alternative splicing, DNA methylation, etc. [5,6,7,8]. This phase polymorphism phenomenon is a very complex multifactorial process. It is of great interest to investigate the gene regulatory mechanisms responsible for locust morphogenesis by experimentally varying rearing densities during nymphal development for probing density-dependent phenotypic plasticity. *Oedaleus asiaticus* is regarded as the most dominant grasshopper species in the range land on the Mongolian plateau of China [9,10]. The locust plagues have not only accelerated grassland degradation and desertification but also have caused severe loss of foliage, posing a serious threat to the livelihood of local farmers and herdspeople [11]. *O. asiaticus* adults have a strong flight capability, up to hundreds of kilometers within a few days under natural conditions, particularly facilitated by high-altitude windborne movements [12]. *O. asiaticus* exhibits clear population density-based phase polyphenism and has two morphs: the darker-brown-colored morph belongs to the high-density population outbreak, while the green morph is a solitary individual [13,14]. 

Population density is considered the most important factor in phase changes in insects. Most orthopteran locusts are known to have density-dependent polytheism, especially in *Locusta migratoria* [15], and show differences in body color, behavior, physiology, and molecular level [16,17]. The solitary individuals are shy, unsociable, and rejected by the same species, while gregarious individuals prefer to gather and migrate in groups as food hunters, posing a greater threat to agriculture [16,18]. Aggregation and migration are the most significant behavioral characteristics of the phase transition from solitary to gregarious individuals in *Locusta migratoria*, while the darkening of body color is the most significant phenotypic characteristic [19]. Similar to migratory locusts, *O. asiaticus* locusts show density-dependent polymorphism under high-density population stress, and their body color changes from green to brown. This phenotypic change is network regulated, involving interactions among multiple elements, including regulatory genes, catalytic enzymes, and signal compounds. 

In the past few years, scientists have used advanced methods and techniques to enhance our molecular understanding of this phenomenon. These investigations and analyses at different levels have provided many novel insights, such the study of *Schistocerca gregaria* identification of novel factors with a possible regulatory role in phase-dependent characteristics or phase transformation, i.e., foraging genes [20], protein kinase A [21], neuropeptide F [22], etc. Insect juvenile hormones have important modulatory roles in phase change, such as mediating the insect neurocentral cascade, which markedly affect insect behavior [23,24,25]. Studies have revealed that the olfactory-related genes *CSP* and *TO* play a key role in mediating the aggregation and repulsion behavior of locusts [26], which are also regulated by juvenile hormones [27]. Further studies in *Locusta migratoria* have shown that neurochemicals play a vital role in solitary to gregarious transition (such as dopamine, serotonin, etc.), mediating the phenotypic changes [28]. Compared to solitary locusts, the brain dopamine level was found to be higher in gregarious locusts, indicating that dopamine induces aggregation behavior during phase changes. In addition, the two dopamine receptors were shown to have opposite regulatory roles in locust aggregation and exclusion behaviors [29]. Meanwhile, to fully understand phase polyphenism, it is important to screen phase-dependent differentially expressed genes (DEGs) and identify their functions.

Several studies have been conducted to elucidate the underlying mechanisms of phase polyphenism in migratory and desert locusts [7,16,30,31]. Large-scale gene expression comparisons between solitary and gregarious phases were performed to find related DEGs [7,32]. However, the underlying molecular mechanism of population density–based phase transformation in *O. asiaticus* is yet unknown. Moreover, *O. asiaticus* is an atypical migratory locust with possible similarities and differences in phase transition compared with other migratory locusts. In this study, we used transcriptome sequencing technology to examine phenotypic and gene expression changes at different time courses during the solitary to gregarious phase transition in locusts under high-density treatment. We mainly focused on how *O. asiaticus* with density-dependent phenotypic plasticity changes body color and gene expression during phase transition and how similar or different it is from other migratory locusts. Understanding the genotypic variation mechanism in *O. asiaticus* can provide a theoretical basis to control its population outbreak to better manage/avoid agriculture loss from locust attack.

## 2. Materials and Methods

### 2.1. Insects Materials and Sampling

*O. asiaticus* locusts were collected from the Siziwangqi grassland of Inner Mongolia, China, in 2019 and reared at the Institute of Grassland Research, Chinese Academy of Agricultural Sciences. The insects were maintained in an incubator (temperature: 25 ± 2 °C; RH: 80 ± 5 °C; Photoperiod: 16 h light: 8 h dark) and fed with fresh wheat and corn leaves twice a day.

Third instar nymphs of solitary *O. asiaticus* with the same developmental status were selected and exposed to high-density gregarious stress. A total of 100 nymphs were collected in each cage and observed to record the insect color change every 12 h. The experiment was set up with 3 equal cages as replicates; there were four treatments after 1, 3, 5, and 7 days of high-density treatment. The individuals whose body color changed from green to brown were randomly selected and treated with liquid nitrogen for extraction of total RNA. Transcriptome sequencing was performed on days 1, 3, 5, and 7 for treated individuals. The control group included individually reared green solitary locusts of the same developmental status. Three individuals from each treatment and control group were used as replicates for sequencing.

### 2.2. RNA Extraction and Construction of cDNA Library

Total RNA was extracted using a Trizol reagent kit following the manufacturer’s instructions. RNA concentration and purity were determined using an Agilent 2100 Bioanalyzer. Quality-qualified RNA samples were used for library construction and sequencing. After the fragmentation of mRNA, random primers were used for reverse transcription to synthesize double-stranded DNA (cDNA). The cDNA was purified with the QIAquick PCR extraction kit (Qiagen, Venlo, The Netherlands), end-repaired, poly(A)-added, and ligated to sequencing adapters. cDNA fragments of preferentially 200 bp in length were selected for PCR amplification to prepare the library, which was sequenced on the Illumina HisSeq platform by the Gene *Denovo* Biotechnology Co. (Guangzhou, China). 

### 2.3. Sequence Assembly and Acquisition of Unigenes

To obtain high-quality clean reads, raw reads were filtered by fastp (version 0.18.0) [33]. Specifically, the reads that contained adapters, >10% of unknown nucleotides (N), and/or failed *Q*-value ≤ 20 bases were removed. Next, the clean reads were assembled by Trinity2.8.4 (https://github.com/trinityrnaseq/trinityrnaseq (accessed on 8 August 2022)) to finish the acquisition of sequences. Unigenes with good integrity were analyzed by BLAST 2.6. (http://ftp.ncbi.nlm.nih.gov/blast/executables/blast+/2.6.0/, accessed on 1 May 2020) and aligned with the reference genome of the databases Nr (NCBI non-redundant protein sequences), SwissProt (a manually annotated and reviewed protein sequence database), KEGG (Kyoto Encyclopedia of Genes and Genomes ortholog database), and KOG (Clusters of Orthologous Groups of proteins) for annotation. The data of unigenes were homogenized for quantification using the RSEM1.2.19 software (accessed on 1 May 2020; http://deweylab.github.io/RSEM/). The FPKM (fragments per kilobase of transcript per million mapped reads) value was used to represent the expression level of corresponding transcripts. Differential expression analysis was performed using the DESeq2 1.20.0 software (http://www.bioconductor.org/packages/release/bioc/html/DESeq.html, accessed on 1 May 2020). The Benjamini and Hochberg approaches were used to adjust *p* values (false discovery rate, FDR) [34,35], and the genes with *FDR* < 0.05 and |log2F| >2 were assigned as DEGs; F represents the fold change in gene expression level [36,37].

### 2.4. Differentially Expressed Gene Enrichment Analysis

GO functional enrichment analysis was performed on the screened DEGs [34,38]. The unigenes annotated by Blast2 GO 2.3.5 (https://www.blast2go.com/, accessed on 1 May 2020) in the GO database were mapped to respective terms, and the number of genes per term was calculated. The number of genes with a certain GO function was obtained, and the hypergeometric test was performed for GO enrichment analysis of DEGs [39,40].

In general, genes interact with each other to perform certain biological functions. A pathway-based analysis helps to understand the biological functions of related genes. KEGG is the major public pathway-related database [22,41]. Therefore, KEGG pathway enrichment analysis was performed to identify significantly enriched metabolic or signal transduction pathways related to DEGs. The calculating formula is the same as that of GO analysis.

### 2.5. Quantitative Real-Time PCR (qRT-PCR)

To validate the seq-data, the expressions of selected induced genes (total 9, *JHAMT* (juvenile hormone acid methyltransferase), *JHEH* (juvenile hormone epoxide hydrolase), *DIB* (ecdysteroid 22-hydroxylase), *HPD* (4-hydroxyphenylpyruvate dioxygenase), *TAT* (Tyrosine aminotransferase), *PAH* (Phenylalanine hydroxylase), *DDC* (DOPA decarboxylase), *CSP* (Chemosensory protein), and *TO* (takeout)) under high-density gregarious stress were determined by qRT-PCR at 1, 3, 5, and 7 days. The gene-specific primers were designed using the Primer 5.0 software. The housekeeping *β-actin* gene was used as the reference gene. A post-amplification melting curve (60 to 95 °C) was performed to complete non-specific product amplification. qRT-PCRs were performed in a 20 μL mixture, consisting of 2 μL cDNA, 0.4 μL forward and reverse primer (10 μmol/L), 10 μL GoScript™ Reverse Transcription Mix, Oligo(dT) (Promega), and 7.2 μL Rnase-free H_2_O. The procedure of qRT-PCR was as follows: initial denaturation at 95 °C for 2 min, followed by 40 cycles at 95 °C for 3 s, 60 °C for 30 s, and lastly 72 °C for 15 s. The qRT-PCR data were analyzed by the 2^−ΔΔCT^ method [42,43] based on three of each biological and technical replicates.

### 2.6. Statistical Analysis

All datasets were analyzed using the SPSS 20.0 software and are presented as mean ± SE (standard error). The difference in gene expressions was analyzed using the one-way analysis of variance (ANOVA) followed by Tukey’s Honestly Significant Difference (HSD) tests (*p* < 0.05).

## 3. Results

### 3.1. High-Density Stress Changes the Body Color of O. Asiaticus

**Figure 1:**
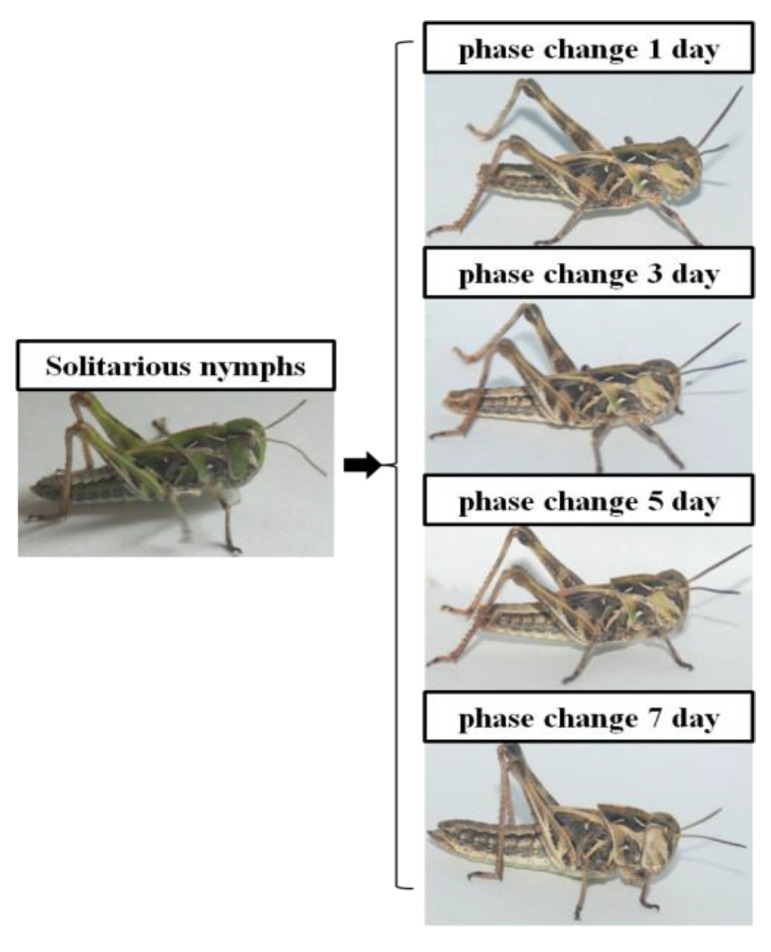
Phenotypic change in *O. asiaticus* on days 1, 3, 5, and 7 (from top to bottom) after high-density treatment.

In general, the body color of solitary individuals of *O. asiaticus* is green, which, upon exposure to high density changed to brown, including the color of legs and pronotum. This phenotypic change began on day 1 of the phase change and was almost completed by the seventh day. The change in body color became more comprehensive with the longer duration of gregarious treatment (Figure 1).

In the high-density population environment (100/cage; cage size: 50 × 50 × 50), the insects began to change body color from green to brown within the first 24 h. The transition was higher at the beginning of treatment; around 6.92 and 7.69% of individuals changed to brown on the first and second days of the treatment, respectively. The cumulative number of transformations reached 67, accounting for 51.53%, on the 12th day of living in a group (Table 1).

### 3.2. Transcriptome Changes in Response to High-Density Treatment

#### 3.2.1. Annotation of Unigenes

**Figure 2:**
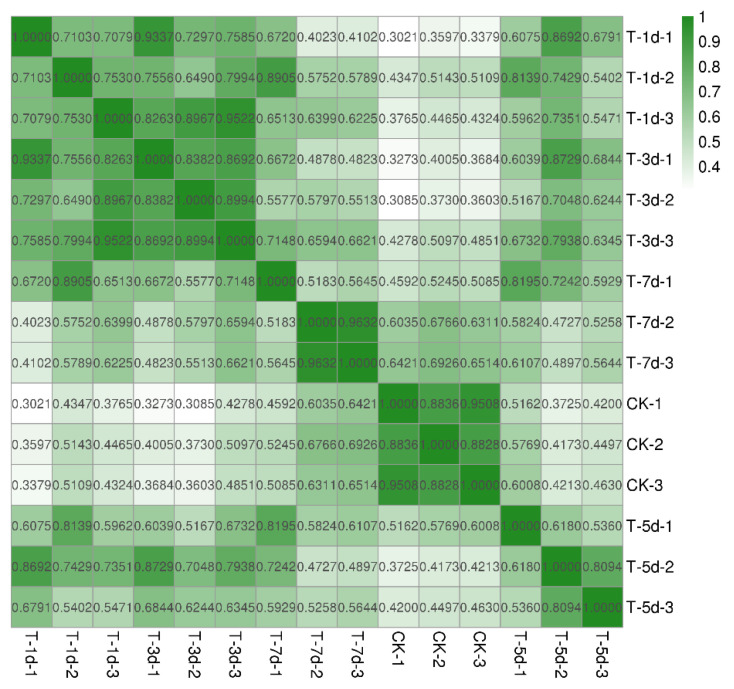
Heat map of differentially expressed genes between different samples; the horizontal and vertical coordinates show the respective sample, and the color shades indicate the magnitude of the Pearson correlation coefficient between the two samples. A color closer to green indicates a higher correlation, while the color closer to white implies a lower correlation. Sample naming rules are CK for control; T for treatment; -1d, -3d, -5d, -7d for day 1, 3, 5 and 7 after phase change; -1, -2 and -3 for biological replicates of different individuals. Combinations are named, e.g., T-1d-1 represents the first biological replicate on day 1 after phase change; CK-1 represents the first biological replicate of the control group.

The transcriptome results yielded a total of 60,502 unigenes with 43.09% GC content and 9159 N50s with an average length of 2015 bp. Before DEGs analysis, the expression of a gene (from the whole gene set) in any two samples was subjected to Pearson correlation coefficients analysis, and the results were presented in the form of heat maps to understand the correlation between any two samples. The Pearson correlation coefficients were higher between the samples of the same treatment and lower between the samples of different treatments, indicating a significant difference between the gene sets of distinct treatments that could be used for the screening of DEGs (Figure 2).

Next, the unigenes were compared with four databases; the Nr database had the most annotated genes (27,820; 45.98%), followed by the KEGG database (21,529; 35.58%) (Table 2). Nr database comparison showed the highest homology of 17.66% with *Zootermopsis nevadensis*, followed by 4.72% with *Gregarina niphandrodes*.

#### 3.2.2. 698 DEGs Were Common to all Four Stages of the Phase Change

**Figure 3:**
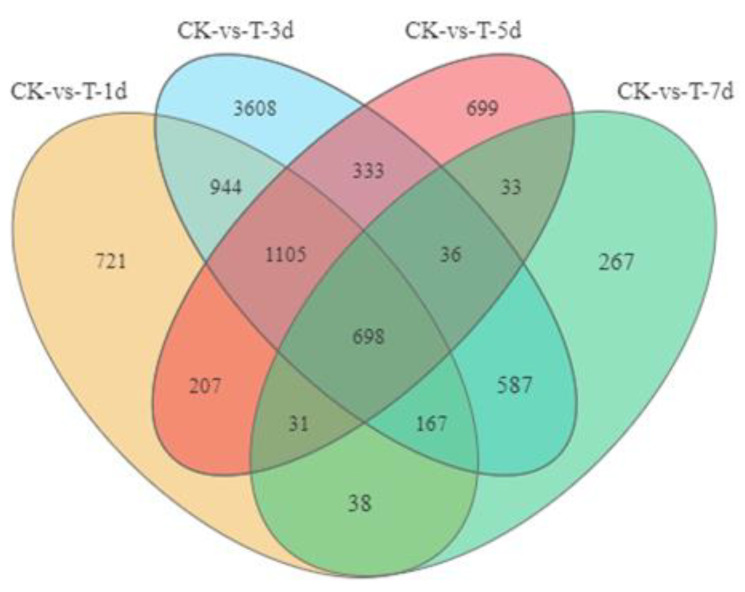
Venn diagram of differentially expressed genes after high-density treatment.

To reveal the molecular mechanism of phase change at the transcriptional level in *O. asiaticus*, RNA-Seq of the adults was performed under high-density treatment. The genes were considered DEGs based on the criteria |log_2_ Fold Change| > 1 and *p* < 0.05. In total, 3911, 7478, 3142, and 1852 DEGs were identified between CK (control) vs. 1d, CK vs. 3d, CK vs. 5d, and CK vs. 7d, respectively. Compared to CK, 2120, 4164, 1343, and 488 genes were upregulated while 1791, 3317, 1799, and 1368 were downregulated in 1d, 3d, 5d, and 7d samples, respectively. The Venn diagram shows that 698 DEGs were common to all four stages of the phase change (Figure 3).

### 3.3. GO and KEGG Enrichment Analyses of DEGs

**Figure 4:**
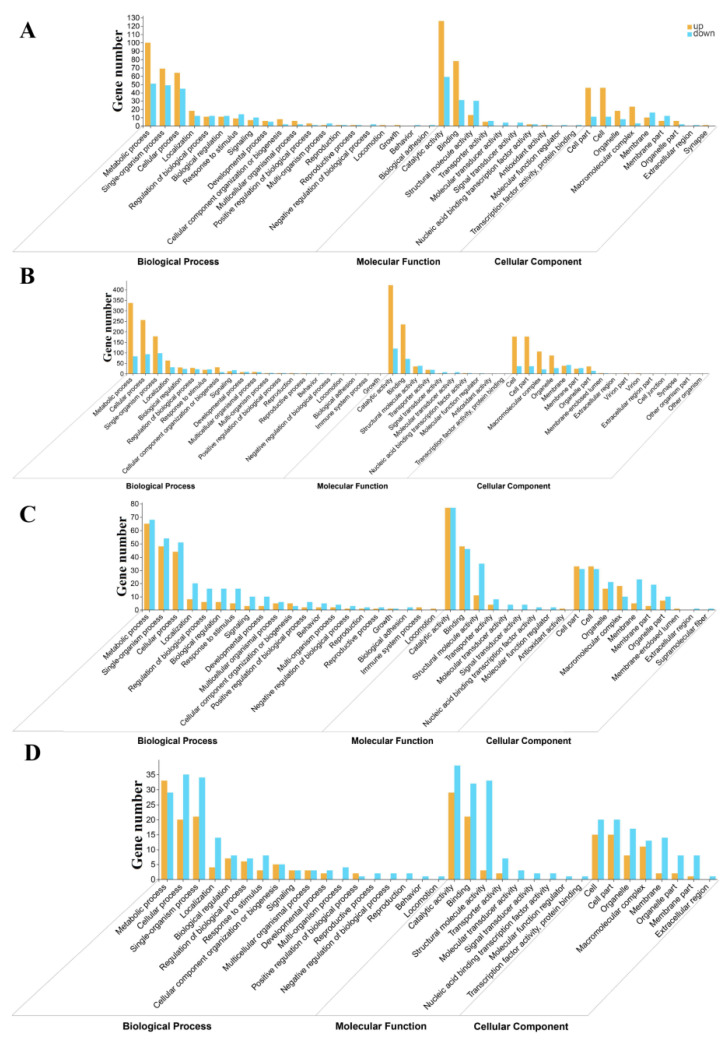
GO enrichment analysis of differentially expressed genes CK (control) vs. 1d (**A**), CK vs. 3d (**B**), CK vs. 5d (**C**), and CK vs. 7d (**D**).

Next, we analyzed GO terms for DEGs in the four stages of *O. asiaticus* phenotypic transformation using the GOseq tool [44]. The GO enrichment analysis revealed that the *metabolism process*, *catalytic activity*, *single-organism metabolism process*, and *small molecule activity* were the most significantly enriched terms for the CK vs. 1d comparison (Figure 4A). For the CK vs. 3d comparison, the most significantly enriched terms were the metabolism process, catalytic activity, pyruvate metabolic process, and organic acid metabolic process (Figure 4B). For the CK vs. 5d comparison, the most significantly enriched terms were the metabolism process, catalytic activity, single-organism metabolism process, and oxidation-reduction process (Figure 4C). For the CK vs. 7d comparison, the most significantly enriched terms were the metabolism process, biding, catalytic activity, organic acid transport, and carboxylic acid transport (Figure 4D).

**Figure 5:**
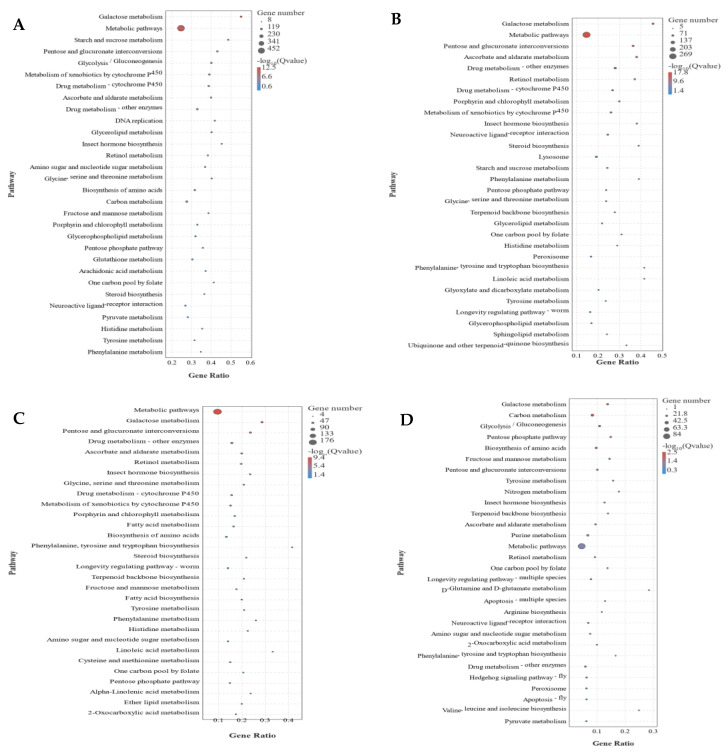
KEGG enrichment analysis of differentially expressed genes CK (control) vs. 1d (**A**), CK vs. 3d (**B**), CK vs. 5d (**C**), and CK vs. 7d (**D**).

To analyze the putative molecular mechanism(s) of phenotypic change under high-density stress, the annotated DEGs were subjected to KEGG enrichment analysis. For CK vs. 1d comparison, 556 DEGs were enriched in 120 KEGG pathways, and the main enrichment pathways included galactose metabolism, metabolic pathways, starch and sucrose metabolism, pentose and glucuronate interconversions, and glycolysis/gluconeogenesis (Figure 5A). For CK vs. 3d comparison, 1133 DEGs were enriched in 130 KEGG pathways, and the main enrichment pathways were galactose metabolism, metabolic pathways, pentose and glucuronate interconversions, ascorbate and aldarate metabolism, and drug metabolism–other enzymes (Figure 5B). For CK vs. 5d comparison, 374 DEGs were enriched in 115 KEGG pathways, and the main enrichment pathways included metabolic pathways, galactose metabolism, pentose and glucuronate interconversions, drug metabolism–other enzymes, and ascorbate and aldarate metabolism (Figure 5C). For CK vs. 7d comparison, 220 DEGs were enriched in 98 KEGG pathways, and the main enrichment pathways were galactose metabolism, carbon metabolism, glycolysis/gluconeogenesis, pentose phosphate pathway, and biosynthesis of amino acids (Figure 5D). These results indicated that the metabolic pathways were mainly affected after high-density treatment of *O. asiaticus*.

### 3.4. qRT-PCR Validation of RNA-seq Data

**Figure 6:**
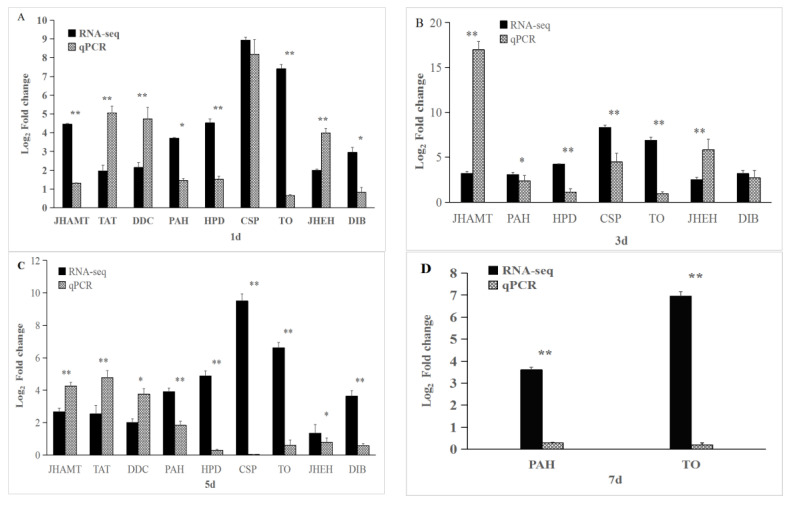
Validation of RNA-seq data by qRT-PCR of the selected differentially expressed genes from the day 1 (**A**), 3 (**B**), 5 (**C**), and 7 (**D**) samples. The gene names are mentioned on the x-axis. The average value of the reference genes (β-actin) was used to normalize transcript levels in each sample. The relative expression level of each gene was calculated by the 2^–∆∆CT^ method. Error bar represents mean ± SE. * means significant difference at *p* < 0.05 level; ** means highly significant difference at *p* < 0.01 level; NS means no significant difference.

To validate the results of RNA-seq, we measured the transcript levels of selected DEGs from 1-, 3-, 5-, and 7-day samples by qRT-PCR. In general, RNA-seq expression patterns were consistent with qRT-PCR results, showing a trivial bias due to the difference between the two methods. These results indicated that the Illumina RNA-seq data were reliable (Figure 6).

### 3.5. Expression Patterns of DEGs

**Figure 7:**
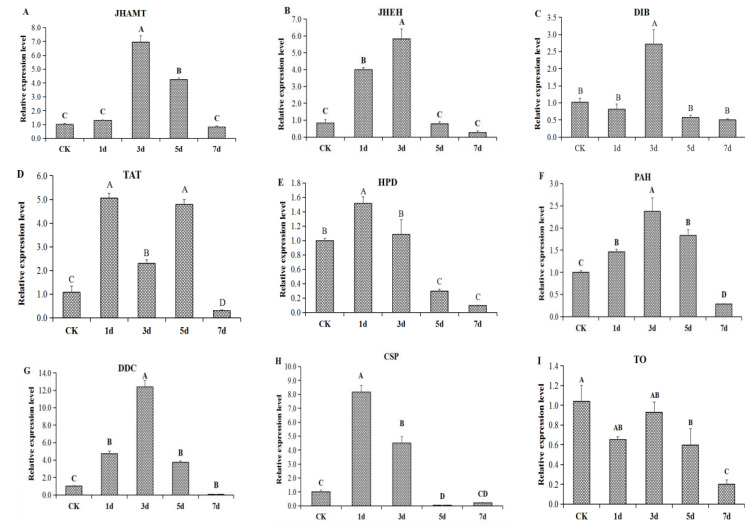
Effect of high-density stress on the expression of selected DEGs at different stages of phenotypic change in *O. asiaticus*. Different letters above the columns indicate significant differences at different time points (*p* < 0.05; Tukey’s HSD test), and data are presented as means ± SD (*n* = 4). *JHAMT* (**A**), *JHEH* (**B**), *DIB* (**C**), *HPD* (**D**), *TAT* (**E**), *PAH* (**F**), *DDC* (**G**), *CSP* (**H**), and *TO* (**I**).

To understand the molecular mechanism of phenotypic change in *O. asiaticus* under high-density stress, a total of nine key candidate genes that regulate the phenotypic change were examined for differential expression. These DEGs are involved in key metabolic pathways regulating insect hormones, dopamine metabolism, and chemosensory proteins. The relative expression profiles of the selected nine signaling-related DEGs, including *JHAMT, JHEH, DIB, HPD, TAT, PAH, DDC, CSP, and TO,* were analyzed by qRT-PCR at different stages. Compared to the controls, *O. asiaticus* exposed to high-density stress showed different expression patterns of hormones, amino acids, and other related factors. For instance, *JHAMT* and *JHEH* related to juvenile hormone anabolism were significantly upregulated on day 3 of the phase change (Figure 7A,B), while *DIB* was upregulated on day 3, showing a significantly higher level than the other days (Figure 7C). *HPD* was upregulated on day 1, showing no difference from the control on day 3, and then became significantly lower on days 5 and 7 (Figure 7D). *TAT* was upregulated on days 1, 3, and 5, showing a significantly higher level than the control group, and then decreased on day 7 (Figure 7E). *PAH* was significantly upregulated on days 1, 3, and 5, and then significantly downregulated on day 7 (Figure 7F). *DDC* was upregulated on days 1, 3, and 5 and then decreased on day 7 (Figure 7G). *CSP* was upregulated on days 1 and 3, decreased on day 5, and then slightly improved on day 7 (Figure 7H). Interestingly, *TO* first increased (days 1 and 3) and then showed a downward trend; however, the levels always remained somewhat lower than the control group (Figure 7).

## 4. Discussion

Several studies have shown that density-dependent phenotypic plasticity is a common phenomenon in insects [13,45,46]. Similar to most locusts, *O. asiaticus* also exhibits distinct density-dependent polymorphism, i.e., under a high-density stress environment, a certain percentage of solitary individuals change into gregarious individuals. During this change, *O. asiaticus* changes body color from green to brown [47,48]. The same was also seen in this report (Figure 1); *O. asiaticus* began with the browning of the head and pronotum from a green body, which continued to other parts over about a week during the phase change. This indicated that high population densities are an important factor for the phenotypic variation in *O. asiaticus*.

Phenotypic plasticity is largely a transcription-based phenomenon, in which small changes in transcript levels produce pronounced changes in phenotype [49]. Here, we investigated the changes in the associated gene regulation network of *O. asiaticus* under high-density treatment using transcriptome sequencing. We found that expressions of several genes (DEGs) changed during the phase change, and these were enriched in various metabolic pathways. This suggested that high-density stress drives phenotypic change in *O. asiaticus* by regulating the related metabolic processes. Importantly, the highest number of DEGs was noticed on day 3 of phase transformation, indicating the peak of the phenomenon by implicating a large number of genes in differential expression. The relatively small number of DEGs at the initial and end stages of the phase transition suggests that phenotypic change is a phasic process, where several genes participate at the onset and then return to normal levels. Notably, the effect of the high-density environment on phenotype change became evident on day 3, and then the DEGs expression started to subside. Notably, most of the DEGs regulating the phenotypic change in *O. asiaticus* also participate in regular activities in both solitary and gregarious individuals, indicating the biological complexity of phenotypic change.

A high-density population environment creates stress for solitary locusts, which respond through a variety of internal stress responses, including changes in behavior, level of physiological substances, immunity, and resilience-related substances [49,50]. In this study, GO and KEGG functional annotation of DEGs showed that these were significantly enriched in anabolic pathways of hormones, phenylalanine, tyrosine, amino acids, and energy substances, and the immunity-associated Toll and Imd signaling pathways. This is consistent with Ma et al. [24], who showed phenotypic variation in the flying locust, where DEGs were enriched in general metabolism-related pathways. This indicates great similarities between the phase-transition process of *O. asiaticus* and other insects, where phenotypic variation leads to individual changes at the physiological levels, and then phenotypic at the morphological levels.

Under hormone imbalance, insect hormones, the regulators of insect growth and development, can effectively change the normal growth and development phenotypic of an insect. Insect hormones also play important roles in phenotypic plasticity, which may also involve many other factors [51,52]. The juvenile hormone, a family of sesquiterpenoids, plays a critical regulatory role in insect metamorphosis, development, pheromones, and body color [53,54]. This study found that the expressions of several key genes involved in JH biosynthesis and degradation (such as *JHAMT* and *JHEH*) were altered under high-density stress. *JHAMT* is the most important regulator of JH biosynthesis and transportation [55,56]. Our qPCR-PCR results showed that *JHAMT* was upregulated on the three days after high-density treatment. Guo et al. [57] used JHAMT knockdown to study its effect on the olfactory-related genes *TO* and *CSP* and found that it increases the expression of *TO* but suppresses the expression of *CSP*. In this study, CSP expression on days 5 and 7 (near the completion of phase transition) showed a similar trend, while the *TO* expression pattern did not correlate. Therefore, the regulatory mechanism of JH on the peripheral olfactory system of *O. asiaticus* needs a detailed study. *JHEH* participates in the degradation of *JH* [13]. We found that high-density treatment promoted the expression of *JHEH* in *O. asiaticus*, which could be related to the reduction of the juvenile hormone during phase change. Since the juvenile hormone is involved in the growth and development of insects, it is also required for regular life activities. Therefore, it makes sense that its level returned to normal near the end of the phase transition. In all, the differential expression of *JHAMT* and *JHEH* indicates their crucial role in solitary to gregarious phenotypic change in *O. asiaticus*.

Locust phase change includes both phenotypic and behavioral changes, involving complex regulations at transcript levels [58]. A study in *L. migratoria* (a desert *locust*) showed that the density-dependent phase change in locusts is characterized by a change in body color; however, the rate of change differs between the solitary and gregarious individuals, and the time required to change insect behavior is much greater than for phenotypic change [27]. Dopamine, an important neurotransmitter for the growth and development of insects, regulates feeding, behavior, learning, and memory and thus plays an important role in phenotypic plasticity [59,60]. Numerous studies have shown that dopamine is predominantly distributed in the locust brain and thoracic ganglia, and during the phenotypic shift, both crowding and isolation treatments cause transient elevations in dopamine levels. Dopamine also functions as a precursor for melanin [3] and therefore can promote the darkening of the insect body during the phase change [61,62]. In this study, expressions of several genes related to the synthesis and metabolism of dopamine were determined at different stages of the phase transition. Notably, phenylalanine and tyrosine are the precursors of dopamine synthesis [63]. Phenylalanine hydroxylase catalyzes the formation of tyrosine from phenylalanine. *PAH*, a key gene of this process, was consistently expressed at high levels on days 1, 3, and 5 of the phenotypic change, indicating the upregulation of tyrosine synthesis. Tyrosine is mainly consumed in the synthesis of (i) acetoacetate catalyzed by tyrosine aminotransferase and (ii) L-DOPA by hydroxylation of tyrosine [64,65]. L-DOPA is further broken down into dopamine and melanin precursors, while dopamine can be further broken down to form yellow pigment. Two important genes of the former pathway, *TAT* and *HPD*, were upregulated during the initial stage of phenotypic transition but returned to normal on day 3. This suggested that tyrosine consumption in this pathway was reduced by day 3, while the other pathway, involving the *DDC* gene for dopamine production by dopamine decarboxylation, began to rigorously consume tyrosine on day 3 of the transformation. Overall, dopamine content was significantly higher on day 3. This is consistent with Ma et al. showing the role of dopamine metabolic pathways in the phenotypic darkening and phase transition of migratory locusts. Dopamine, an important regulatory metabolite, plays a regulatory role in the aggregation behavior and phenotypic (body color) change of locusts.

In all, we showed that phase transition in *O. asiaticus* is regulated by the genes related to juvenile hormones and dopamine. Population density stress drives phenotypic change in *O. asiaticus* by regulating morphological, phenotypic, and metabolic changes involving complex interactions among different regulatory pathways at multiple levels.

## 5. Conclusions

(i) Phenotypic changes in *O. asiaticus* at high densities demonstrate that population density is an important inducing factor in the transition from solitary to gregarious in *O. asiaticus*; (ii) phenotypic changes in *O. asiaticus* are a continuous process that takes time; (iii) phase changes are evident at both the phenotypic and genetic levels; (iv) the phenotypic transition from solitary to gregarious occurs first by a local change in body color, and only after a certain period of time does the body color change completely. In the genetic aspect, the number of DEGs did not change significantly at the beginning of the phenotypic change process but was consistent with the phenotypic change. The number of DEGs increased significantly at the middle of the phenotype change, when the phenotype change was most obvious, and the qPCR results showed that a large number of genes had relatively high expression at 3 days, while at the end of the phase change, the number of DEGs decreased significantly, and the expression largely returned to normal levels, indicating that the phenotype change of *O. asiaticus* was regulated by a large number of genes and that genes regulated the phenotype change through changes in expression. However, the differential changes in expression occurred during the phenotype change and did not persist after the phase change, further suggesting that genes are involved in regulating the phenotype change in *O. asiaticus.*

## Figures and Tables

**Table 1:** Number and proportion of locust gregarious 12-day phenotype variation statistics.

High-Density Treatment Time (d)	Number of Insects Changed to Brown (out of 100)	Proportion (%)
1	9	6.92
2	10	7.69
3	6	4.61
4	6	4.61
5	4	3.08
6	5	3.85
7	4	3.08
8	7	5.38
9	6	4.61
10	5	3.85
11	4	3.08
12	1	0.77
total	67	51.53

**Table 2:** Summary of *O. asiaticus* data annotation from four databases.

Annotated Database and Unigenes	Numbers of Unigenes	Proportion (%)
Nr	27,820	45.98
KEGG	21,529	35.58
COG	14,346	23.71
SwissProt	15,154	25.05
Annotated genes	27,995	46.27
Not-annotated genes	32,507	53.73
Total unigenes	60,502	100

## Data Availability

Not applicable.
